# Hybrid Printing Metal-mesh Transparent Conductive Films with Lower Energy Photonically Sintered Copper/tin Ink

**DOI:** 10.1038/s41598-017-13617-4

**Published:** 2017-10-16

**Authors:** Xiaolian Chen, Xinzhou Wu, Shuangshuang Shao, Jinyong Zhuang, Liming Xie, Shuhong Nie, Wenming Su, Zheng Chen, Zheng Cui

**Affiliations:** 1 School of Nano-Tech and Nano-Bionics, University of Science and Technology of China, Hefei, 230026 People’s Republic of China; 2 Printable Electronics Research Centre, Suzhou Institute of Nano-Tech and Nano-Bionics, Chinese Academy of Sciences, Suzhou, 215123 People’s Republic of China

## Abstract

With the help of photonic sintering using intensive pulse light (IPL), copper has started to replace silver as a printable conductive material for printing electrodes in electronic circuits. However, to sinter copper ink, high energy IPL has to be used, which often causes electrode destruction, due to unreleased stress concentration and massive heat generated. In this study, a Cu/Sn hybrid ink has been developed by mixing Cu and Sn particles. The hybrid ink requires lower sintering energy than normal copper ink and has been successfully employed in a hybrid printing process to make metal-mesh transparent conductive films (TCFs). The sintering energy of Cu/Sn hybrid films with the mass ratio of 2:1 and 1:1 (Cu:Sn) were decreased by 21% compared to sintering pure Cu film, which is attributed to the lower melting point of Sn for hybrid ink. Detailed study showed that the Sn particles were effectively fused among Cu particles and formed conducting path between them. The hybrid printed Cu/Sn metal-mesh TCF with line width of 3.5 μm, high transmittance of 84% and low sheet resistance of 14 Ω/□ have been achieved with less defects and better quality than printed pure copper metal-mesh TCFs.

## Introduction

Printed electronics is a new emerging filed in recent years^1,2^. Compared with conventional vacuum deposition and photolithographic techniques, printing is low-cost, efficient for material usage^3,4^, has been applied to fabricated flexible displays, radio frequency identification tags (RFID)^5,6^, and wearable electronics^7,8^.

Conductive ink based on silver nanoparticle is widely used in printed electronics^9,10^, albeit at high cost^11^. There have been continuous efforts in developing copper ink, either based on copper or copper oxide nanoparticles, because of the low price of copper. However, copper nanoparticles are too easily to oxidize and cannot be sintered by traditional sintering methods under ambient condition^12,13^. The photonic sintering using intensive pulse light (IPL) has proved to be effective for copper nanoparticle ink^14,15^. However, the high photonic sintering energy, which is needed to make copper nanoparticle films conductive, leads to the destruction of samples^16^.

One of the important applications of conductive inks is to print conductive electrodes. There has been a considerable surge of activity in the development of printing metal grids as an alternative to ITO for transparent conductive films (TCFs)^17–20^. Among the many reported techniques, the authors’ group developed a hybrid printing process which prints conductive ink into a narrow trench instead of conventionally on surface (The hybrid printing process is shown in Supplementary Fig. S1). In this innovative process, less than 3 μm grid width can be achieved and the embedded Ag metal-mesh TCF has extremely low sheet resistance (<0.5 Ω/□) while still maintains high transparency (>85%)^21^. The hybrid printed TCFs have been successfully used in touch-screen panels and solar cells^21–23^. The authors’ group also conducted research on copper ink and used an IPL for photonic sintering^24^. Unlike Ag ink, Cu ink usually needs very high energy photonic sintering to convert copper oxide into copper. Though high energy photonic sintering poses no problem for copper ink printed on a flat surface, it is problematic if the copper ink is embedded into a trench. Defects in the forms of broken mesh lines and missing copper materials in the trenches happened in the process.

In this study, a Cu/Sn hybrid ink has been developed by mixing Cu and Sn particles. The sintered energy of Cu/Sn film with mass ratio of 2:1 (Cu:Sn) was found 21% less than that for Cu film, which is attributed to the lower melting point of Sn for hybrid ink. The SEM inspection revealed that the molten Sn particles were fused among Cu particles and formed conducting paths on Cu/Sn hybrid film. Using the Cu/Sn ink and previously developed Cu ink at the authors’ group for comparison, metal-mesh TCFs were fabricated by the hybrid printing process and sintered with an IPL. The Cu/Sn metal-mesh TCF achieved the transmittance of 84% and low sheet resistance of 14 Ω/□ with less defects and better quality than printed pure copper metal-mesh TCFs.

## Results

### Photonic sintering of screen-printed Cu and Cu/Sn films

**Figure 1:**
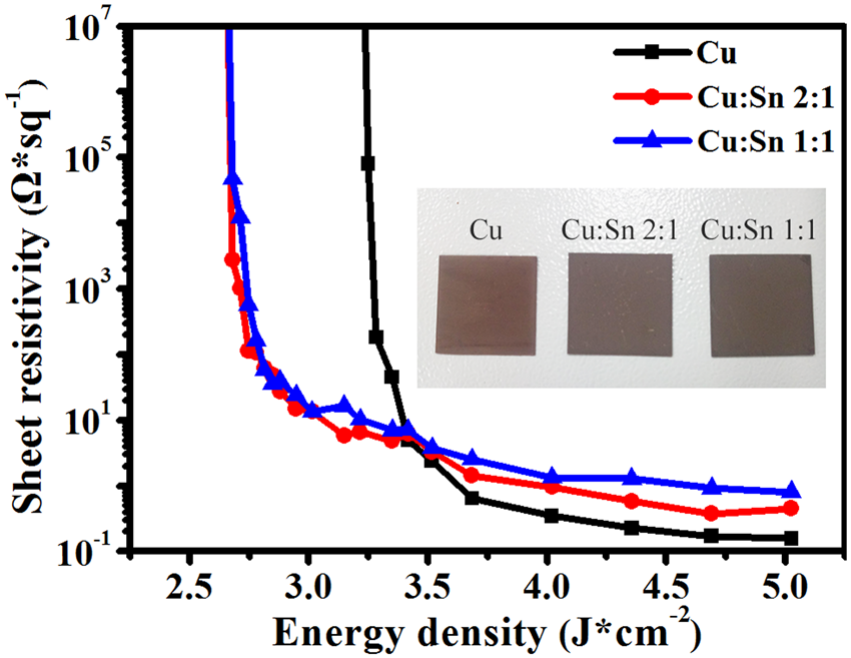
Sheet resistance of Cu and Cu/Sn film (2:1 and 1:1) with respect to different sintered energy density of 2.64 to 5.02 J/cm^2^, (inset) the sintered screen-printed metal films.

In order to study the sintering characteristics of Cu/Sn hybrid film and to compare with the sintering of pure Cu film, the Cu and Cu/Sn films with the size of 2 cm * 2 cm (as shown in the inset of Fig. 1) were fabricated by screen-printing and the IPL energy density ranging from 2.64 to 5.02 J/cm^2^ was applied. Figure 1 presented the sheet resistance of sintered Cu and Cu/Sn hybrid film with respect to irradiation energy. Cu/Sn films of mass ratio 2:1 and 1:1 (Cu:Sn) sintered by 2.68 J/cm^2^ exhibited weak conductivity. And sheet resistance was rapidly down with the increase of sintered energy density. However, the Cu film was non-conductive until sintered by 3.25 J/cm^2^. It implied that the sintering energy density of Cu/Sn hybrid films at the mass ratio of 2:1 and 1:1 was about 21% lower than that of Cu film in order to achieve conductivity. It was also observed that the Cu/Sn films reached to the lowest and stable sheet resistance (2:1: 0.379 Ω/□, 1:1: 0.808 Ω/□) when energy was higher than 3.25 J/cm^2^; but Cu film only moved towards flatness after 3.65 J/cm^2^ and had the lowest sheet resistance of 0.158 Ω/□. The thickness of Cu and Cu/Sn films was analyzed by step profiler (Supplementary Fig. S6). The data presented that Cu and Cu/Sn films before sintering had the similar average thickness, about 6 μm. Meanwhile, the thickness of the Cu and Cu/Sn films decreased to ∼5 μm after the 4.69 J/cm^2^ flash light-sintering process.

**Figure 2:**
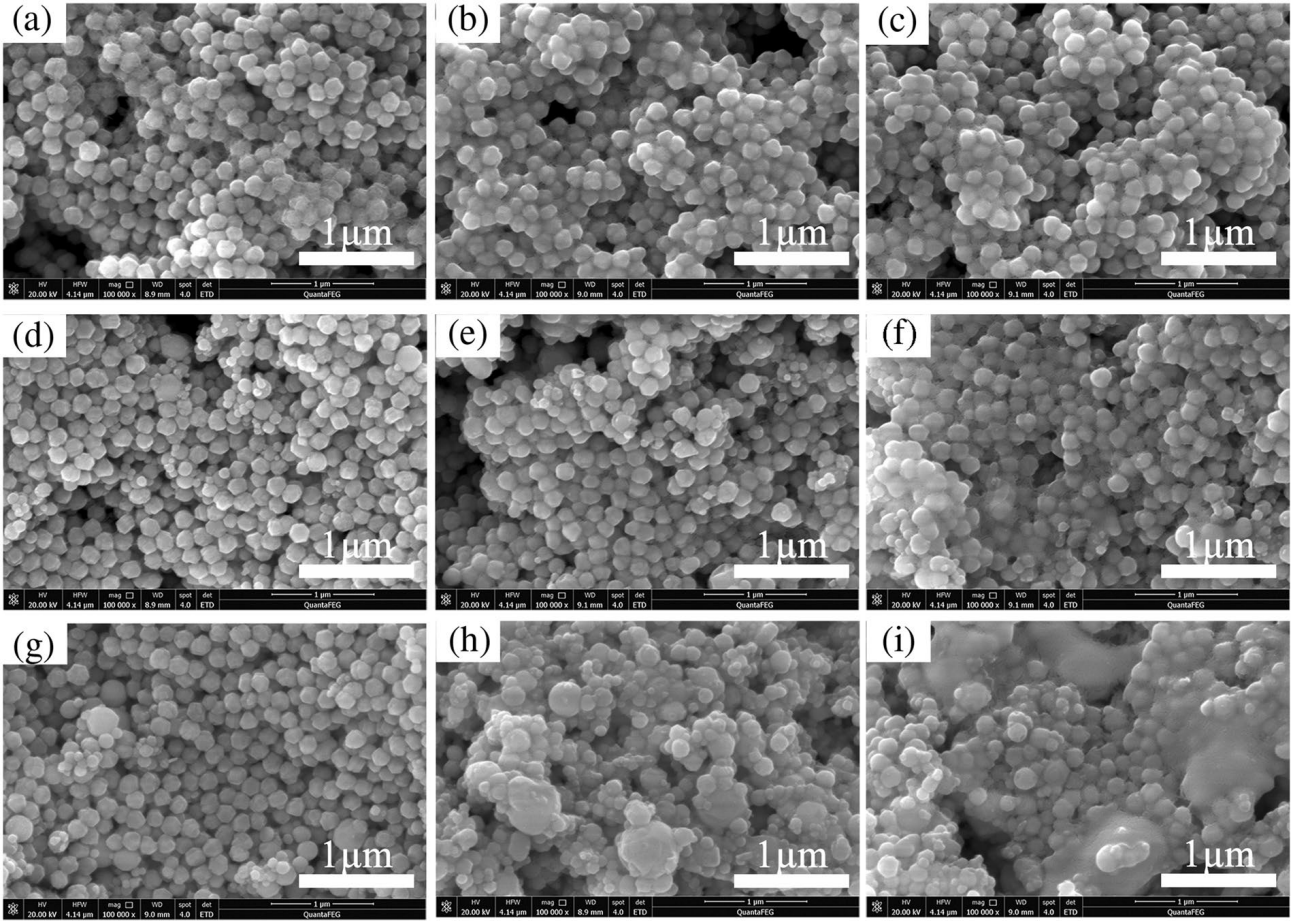
SEM images of Cu and mixed Cu/Sn films before and after sintering by IPL. The top row images (**a**, **b**, **c**) are pure Cu ink, the central row (**d**, **e**, **f**) a 2:1 mixture and the bottom row (**g**, **h**, **i**) a 1:1 mixture. The left column (**a**, **d**, **g**) correspond to samples before sintering, those in the central column (**b**, **e**, **h**) to 3.25 J/cm^2^ and those in the right column (**c**, **f**, **i**) to 4.69 J/cm^2^.

**Figure 3:**
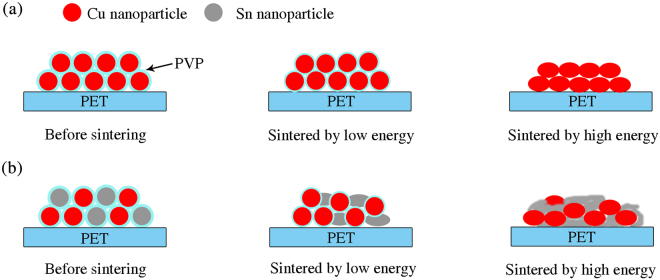
Sintered schematic diagram of Cu and mixed Cu/Sn films under low energy and high energy: (**a**) Cu ink film; (**b**) Mixed Cu/Sn ink film.

Generally, electrical resistance of films made by particle ink is dependent on the intrinsic resistance of metal and contact resistance between metal particles^25,26^. Before sintered by IPL, the surface of spherical Cu and Sn particles were covered by insulating PVP layer and electric charge could not hop into adjacent particles, resulting in non-conductivity in Cu and Cu/Sn films. Figure 2 are the SEM images of Cu and mixed Cu/Sn films before and after sintering by IPL. The top row images (a, b, c) are pure Cu ink, the central row (d, e, f) a 2:1 mixture and the bottom row (g, h, i) a 1:1 mixture. The left column (a, d, g) correspond to samples before sintering, those in the central column (b, e, h) to 3.25 J/cm^2^ and those in the right column (c, f, i) to 4.69 J/cm^2^. At low photonic energy, Sn particles started to melt because of its low melting point (the bulk Sn is 231.89 °C)^27^ and to connect with Cu particles (Fig. 2e,h). Simultaneously, PVP at the surface of particles was decomposed partially and became thinner^28^, as described in schematic diagram of Fig. 3. The charges were able to transport more easily through the interface between Cu particles than that for Cu film, so was the better conductivity of Cu/Sn films than Cu films under the low energy. As the sintering energy density increased, Sn particles were melt completely and Cu particles covered by PVP were also gradually reduced to pure Cu (Fig. 2f,i). The barrier of charge transport between particles for Cu/Sn films was further reduced. Meanwhile, sintered Cu particles on pure Cu film were also connected with each other^14^, so that the charge of Cu film could transport between the melt Cu particles just like the bulk Cu. Owing to the greater conductivity of bulk Cu (1.68 μΩ·cm) than bulk Sn (10.1 μΩ·cm)^27,29^, the conductivity of Cu film sintered by higher energy density were better than Cu/Sn particle ink films, as shown in Fig. 1. Figure S7 in Supplementary Information showed the EDS mapping images of Cu/Sn film (1:1) before and after sintering, which are provided in the Cu (green) and Sn (red) distribution. These images confirmed that the Cu/Sn ink was well-distributed.

**Figure 4:**
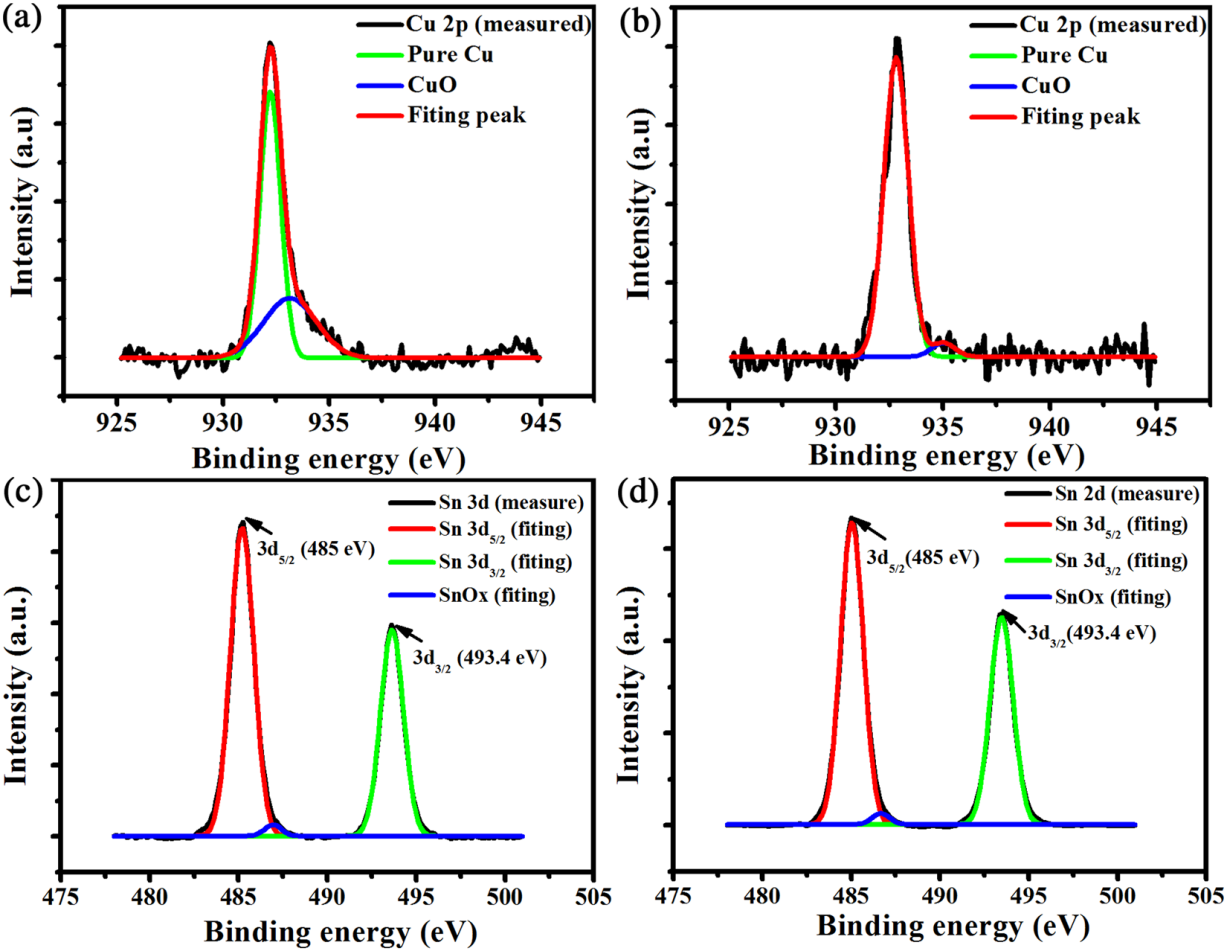
XPS spectra of Cu and Sn particles before and after sintering: (**a**) Cu and (**c**) Sn particles before sintering; (**b**) Cu and (**d**) Sn particles after sintered by 4.69 J/cm^2^.

In order to verify that oxidized Cu particles were reduced to pure Cu particles at the high energy density of 4.69 J/cm^2^, XPS analysis before and after sintering were conducted. As showed in Fig. 4a,b, curve fitting with Gaussian function divided Cu 2p_3/2_ region with two peaks around 932.6 eV and 934.6 eV, which resulted from Cu and CuO, respectively^30^. Before sintering, there was a small Cu 2p_3/2_ peak around 934.6 eV (Fig. 4a). But Cu 2p_3/2_ peak around 934.6 eV nearly disappeared after sintering of 4.69 J/cm^2^ (Fig. 4b), manifesting CuO did not exist on the surface of Cu particles and was totally reduced to Cu by the decomposition of PVP^28^. So the Cu particles on Cu and Cu/Sn films were sintered completely by 4.69 J/cm^2^. At the same time, the XPS analysis of Sn particles was carried out to measure the surface composition (Fig. 4c,d). The Sn spectrum showed two peaks due to spin-orbital coupling of the 3d state: Sn 3d_5/2_ at 485 eV and Sn 3d_3/2_ at 493.4 eV^11^. As shown in Fig. 4c,d, regardless of the flash light-sintering process, the Sn 3d_5/2_ region was divided into two peaks: one strong peak has a binding energy of 485 eV (Sn), another extremely weak peak was around 487 eV, indicating SnO_x_^11,31^. This data indicated that Sn particles was barely oxidized during the IPL process.

**Figure 5:**
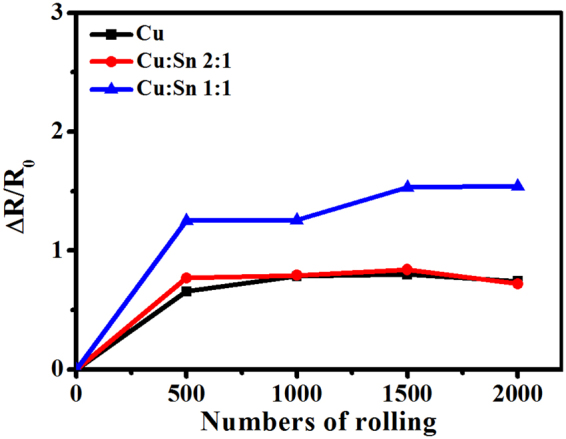
Rolling fatigue test results of Cu and mixed Cu/Sn films.

To compare the mechanical flexibility of sintered Cu and Cu/Sn films, rolling tests were conducted. Figure 5 presented the sheet resistance change about sintered Cu/Sn hybrid films after rolling (ΔR/R_0_). All the films in the rolling test were sintered at 4.69 J/cm^2^. After 500 cycles, ΔR/R_0_ for Cu film was 0.65 while the values for Cu/Sn hybrid films of 2:1 and 1:1 were 0.76 and 1.25, respectively, implying that the sheet resistance just changed a little and exhibited good property of mechanical stability. It was noteworthy that ΔR/R_0_ went up when the mass ratio of Sn in Cu ink was increased. This phenomenon was attributed to that fully melt Sn particles under high energy were fused with Cu particles together (as presented in Fig. 2i) while Cu particles for Cu film could not be fused completely (Fig. 2c). But ΔR/R_0_ for both films retained constant after 2000 cycles and just kept below 1.5, showing good mechanical stability.

### Photonic sintering of hybrid printed Cu and Cu/Sn metal-mesh TCFs

Both the Cu and Cu/Sn ink were applied to hybrid printing of metal-mesh TCFs. Due to faster heat dissipation compared with the scree-printing continuous films, it was found that the higher sintering energy was needed for the narrower Cu grids (3.5 μm) of metal-mesh TCF to achieve the melting temperature of Cu particles^32^. As a result, the energy density of 4.66–10 J/cm^2^ was applied by experimental optimization, using one pulse with fixed 2.6 kV. But many defects and fractured Cu grids were observed on Cu metal-mesh TCF (as showed in Supplementary Fig. S10) because of massive heat and stress concentration.

**Figure 6:**
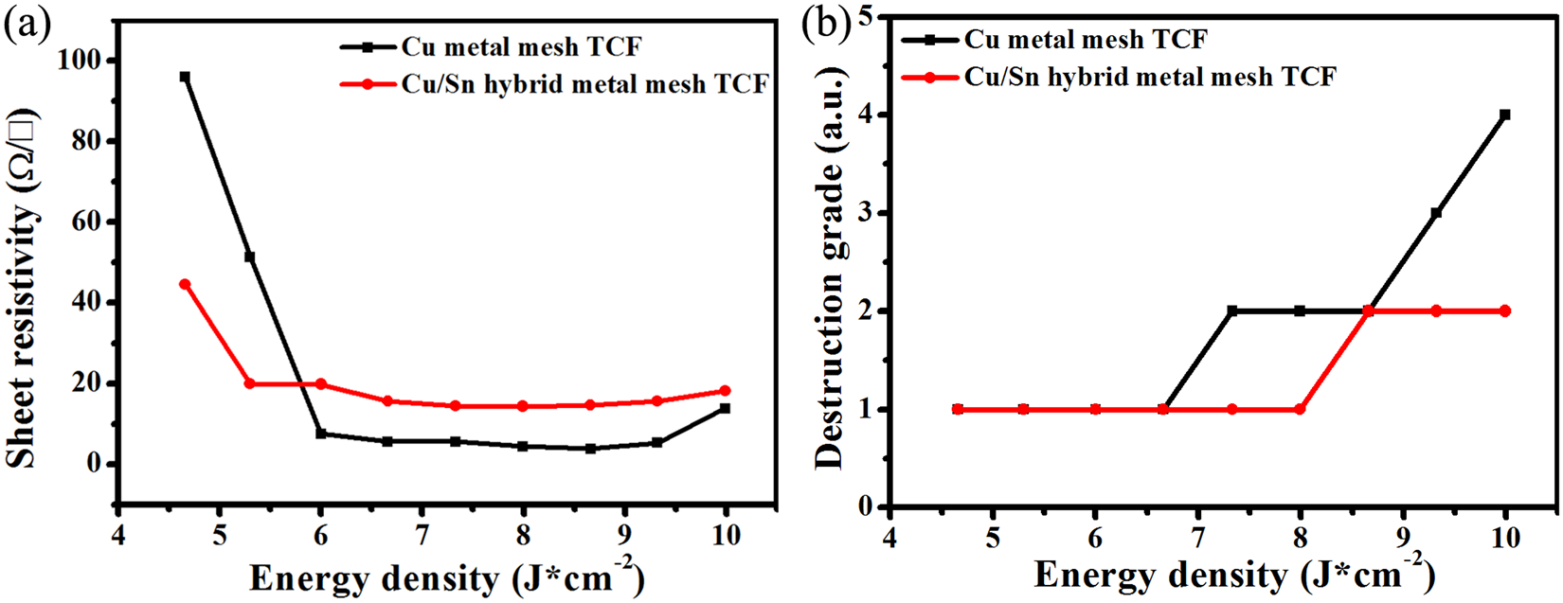
(**a**) The sheet resistance and (**b**) Destruction grade of Cu and Cu/Sn hybrid metal-mesh TCFs sintered by different energy intensity.

In contrast to the hybrid printed Cu metal-mesh, the Cu/Sn particle ink needs lower sintering energy as discussed above. Figure 6a showed the sheet resistance values plotted across the energy density of between 4.66 J/cm^2^ and 10 J/cm^2^. We found that the sheet resistance was lying on the decreasing curve before 8.66 J/cm^2^. And Cu metal-mesh TCF, which had the transmittance of about 84% at 550 nm (Supplementary Fig. S3), had the lowest sheet resistance of 3.8 Ω/□. However, the conductivity would fall off while sintered energy was higher than 8.66 J/cm^2^. For Cu/Sn metal-mesh TCF, it reached the lowest sheet resistance of 14.5 Ω/□ when sintered by 7.33 J/cm^2^ and kept almost invariable after 7.33 J/cm^2^.

Though the conductivity of hybrid printed Cu/Sn metal-mesh TCF was not as advantageous as the Cu metal-mesh TCF, the great benefit of lowering photonic sintering energy is the reduced defect level in the TCFs. To quantify the defect level of TCF, the quality of film was evaluated using a grade scheme^33^. Grade 1 indicates perfectly intact structures in all parts of the test pattern. Grade 2 indicates minor fractured lines. Grade 3 indicates destructive lines and structures below 5% of the whole area. Grade 4 indicates destructive lines and structures at 5% to 20% of the whole area. Grade 5 indicates major damage that make it difficult to measure the sheet resistance. The optical microscope images for different grades were exhibited in Supplementary Fig. S11.

**Figure 7:**
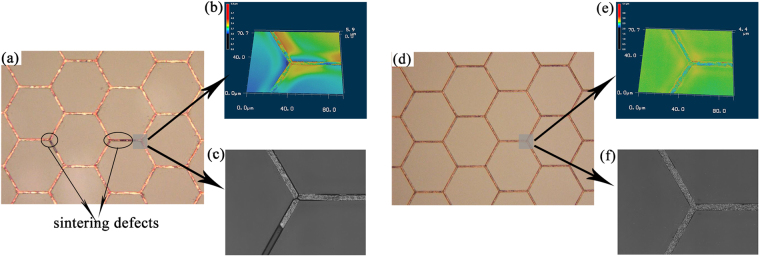
The optical microscope images of metal-mesh TCF sintered by 7.99 J/cm^2^: (**a**), (**b**) and (**c**) Cu metal-mesh TCF; (**d**), (**e**) and (**f**) Cu/Sn metal-mesh TCF; (**a**) and (**d**) In laser and color modes; (**b**) and (**e**) In 3D mode; (**c**) and (**f**) In laser mode.

Figure 6b exhibited the relationship between the film grade and sintering energy. Cu metal-mesh TCF do not show any damage before sintering energy density of 6.66 J/cm^2^. After 7.33 J/cm^2^, the structure showed some damages, and the higher energy density was, the more significant the destruction was. For Cu/Sn metal-mesh TCF (1:1), it displayed excellent film quality before 7.99 J/cm^2^ and just a slight damage after 8.66 J/cm^2^. Figure 7 also presented the optical microscopic images of Cu and Cu/Sn metal-mesh TCF sintered by 7.99 J/cm^2^. The plastic substrate for Cu metal-mesh TCF was deformed and Cu lines in the trenches were broken while Cu/Sn metal-mesh TCF exhibited perfectly intact structure. The much reduced damage was probably the result that the melting of Sn particles consumed additional heat, limiting the maximum temperature. The DSC was analyzed for particles to support the speculation. As shown in Supplementary Fig. S9, the DSC curves of Sn and mixed Cu/Sn ink presented the endothermic peak around 230 °C while it did not exist in the Cu ink, which means that the Cu metal-mesh TCF has higher instantaneous temperature than Cu/Sn metal-mesh TCF under the same condition, causing unreleased massive stress^32,34–37^. On the other hand, the melting of Sn particles around Cu particles played the role of releasing part of stress caused by momentary temperature. Consequently, Cu metal-mesh TCF was damaged more easily while Cu/Sn metal-mesh TCF was not.

**Figure 8:**
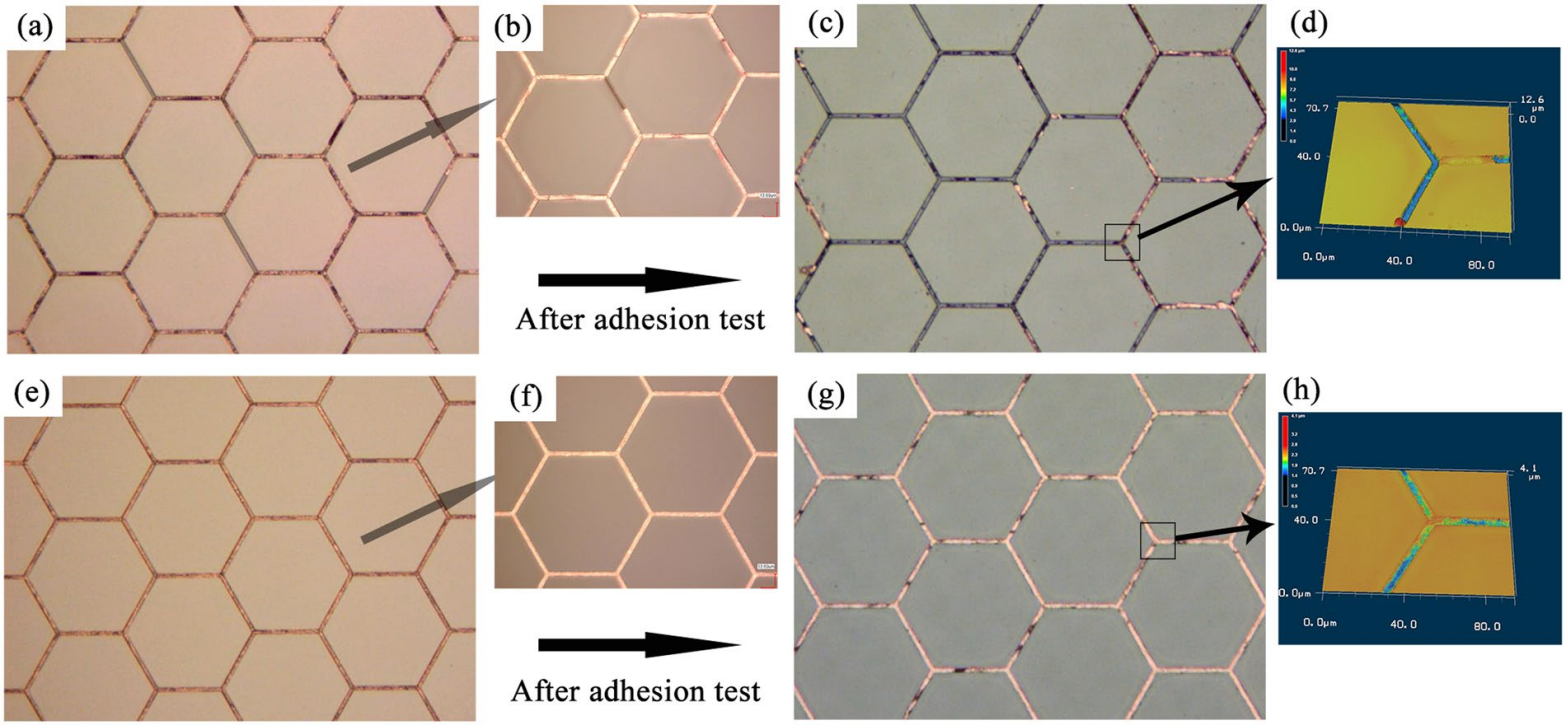
The optical microscope images of metal-mesh TCFs before and after adhesion test: the top row of images showed Cu metal-mesh TCF sintered by 7.99 J/cm^2^; the bottom row showed Cu/Sn metal-mesh TCF; (**a**), (**b**), (**e**) and (**f**) before adhesion test; (**c**), (**d**), (**g**) and (**h**) after adhesion test.

Figure 8 showed the result of adhesion test on metal-mesh TCFs according to ASTM D3359 standard. Figure 8a,b presented the images of Cu metal-mesh TCF sintered by 7.99 J/cm^2^ before adhesion test. After the adhesion test, most of the Cu fell off the trenches, and only 10% of Cu particles left (Fig. 8c,d), resulting in no conductivity. Figure 8e,f are the images of Cu/Sn metal-mesh TCF before adhesion test. After adhesion test, more than 90% of Cu/Sn were still in the trenches (Fig. 8g,h), resulting in the TCF still conductive at the sheet resistance of 28 Ω/□. The Cu grids actually protruded out of the surface due to heat induced stress while there was no noticeable protruding for Cu/Sn metal-mesh TCF (Supplementary Fig. S2). As a result, stronger adhesion of Cu/Sn than Cu only metal-mesh TCF was achieved.

## Discussion

A Cu/Sn hybrid ink has been developed by mixing Cu and Sn particles. The hybrid ink requires lower sintering energy than normal copper ink and has been successfully employed in a hybrid printing process to make metal-mesh transparent conductive films (TCFs). The sintering energy of Cu/Sn hybrid films with the mass ratio of 2:1 and 1:1 (Cu:Sn) were decreased by 21% compared to sintering pure Cu film, which is attributed to the lower melting point of Sn for hybrid ink. Detailed study showed that the Sn particles were effectively fused among Cu particles and formed conducting path between them. The hybrid printed Cu/Sn metal-mesh TCF with line width of 3.5 μm, high transmittance of 84% and low sheet resistance of 14 Ω/□ have been achieved with less defects and better quality than printed pure copper metal-mesh TCFs.

## Methods

### Preparation of Cu/Sn hybrid inks

To fabricate Cu/Sn hybrid inks, commercially available Cu particles with oxide shells (150 nm ± 30 nm in diameter, oxide thickness <3 nm; Suzhou NanoGrid Technology Co. Ltd) and Sn particles (100–300 nm in diameter; Sigma-Aldrich) were used in this study, as shown in Supplementary Fig. S4. Polyvinyl pyrrolidone (PVP, Mw 44000–54000) as the capping agent and ethyl cellulose used as adhesion agent were purchased from Sinopharm chemical reagent Co. Ltd. And Cu and Sn particles were dispersed in a mixed solvent containing 10 g of terpilenol, 1 g of PVP and 0.6 g of ethyl cellulose to fabricate Cu ink and Sn ink, respectively. Then Cu ink was mixed with different mass of Sn ink by stirring for 30 min, followed by ball milling for 8 h. Cu/Sn hybrid inks have different mass ratios of Cu to Sn, about 1:0, 2:1 and 1:1, respectively, as showed in Table 1.

**Table 1:** Contents of formulated Cu/Sn hybrid ink.

Terpilenol (g)	PVP (g)	Ethyl cellulose (g)	Cu particles (g)	Sn particles (g)	Cu:Sn
10	0.8	0.3	16.70	0.00	1:0
10	0.8	0.3	11.13	5.57	2:1
10	0.8	0.3	8.35	8.35	1:1

### Sintering of Cu and Cu/Sn hybrid ink by intensive pulse light (IPL)

Cu and Cu/Sn hybrid inks were screen-printed on a polyethylene terephthalate (PET) substrate with a thickness of 188 μm to fabricate Cu and Cu/Sn hybrid films, followed by drying on a hot box at 80 °C for 2 min. IPL from a xenon flash lamp system (Sinteron 2010, Xenon Corp.) was applied with A type lamp, of which wavelengths ranged from 370–800 nm. The distance from the lamp to substrate stage was 3.5 cm. The sintered energy density of Cu and Cu/Sn hybrid films from 2.68 J/cm^2^ to 5.02 J/cm^2^ was used with the single pulse. It was adjusted by increasing the single pulse time with constant voltage of 2.3 kV.

After sintering, the sheet resistance of Cu or Cu/Sn hybrid films was measured by four-point probe station (Suzhou Jingge Electronic Co., Ltd). The microstructure, surface and elemental mapping of films were tested by scanning electron microscopy (SEM, Quanta 400 FEG). The absorption spectra of Cu and Sn NPs were analyzed with a UV-visible spectrometer (Lambda 750, PerkinElmer).The surface profiles were analyzed by using step profiler (Dektak XT, Bruker Corporation). Differential scanning calorimetry (DSC) was performed Netzsch DSC F3 Maia (Germany) at a heating rate of 10 °C min^−1^ from room temperature to 270 °C under a nitrogen atmosphere.

The mechanical property of sintered Cu/Sn ink films was measured by rolling radius of 10 mm; all kinds of conducting films in rolling test were sintered by 4.69 J/cm^2^ and the sheet resistance change after rolling was determined by:

1 $${\rm{\Delta }}{\rm{R}}/{{\rm{R}}}_{{\rm{0}}}=({\rm{R}}-{{\rm{R}}}_{0})/{{\rm{R}}}_{0},$$

R_0_ was the initial sheet resistance, and R was the sheet resistance measured after rolling.

### Fabrication and sintering of Cu and Cu/Sn metal-mesh TCFs

The Cu and Cu/Sn metal-mesh TCFs were prepared by our proprietary work^21^, and the fabrication process was presented in Supplementary Fig. S1. Firstly, the imprinting technology was used with the imprinting Ni master plate to form the mesh pattern on PET. Then Cu and Cu/Sn hybrid inks were filled into the trenches by blading. Finally, metal-mesh TCF was dried on a hot box at 80 °C for 2 min. The mesh pattern had a transmittance of 91% at 550 nm (as showed in Supplementary Fig. S3). Hexagonal trench structures were 3.5 μm in line width, 3 μm in depth and 74 μm in side length (Supplementary Fig. S5). By blading particle ink, the height of the metal grid was about 2.5–2.7 μm. The sintered energy density of Cu and Cu/Sn metal-mesh TCF from 4.66 J/cm^2^ to 10 J/cm^2^ was used, which was adjusted by changing the single pulse time with constant voltage of 2.6 kV. The sheet resistance of sintered Cu and Cu/Sn hybrid metal-mesh TCFs was measured by four-point probe station. Metal-mesh TCFs were characterized by the optical microscope (color 3D laser scanning microscope VK-9710, KEYENCE). The adhesion of Cu or Cu/Sn metal-mesh TCFs after sintering was tested according to the ASTM D3359 standard (An international standard)^24^.

## Electronic supplementary material

Supplementary Information
