# Supplementary material for: Hybrid Printing Metal-mesh Transparent Conductive Films with Lower Energy Photonically Sintered Copper/tin Ink
Source: Sci Rep. 2017 Oct 16;7:13239. doi: 10.1038/s41598-017-13617-4 (PMC5643557; doi:10.1038/s41598-017-13617-4)
Supplement: Supplementary file 1 — Supplementary Information [file 41598_2017_13617_MOESM1_ESM.doc]

Supporting information

**Hybrid Printing Metal-mesh Transparent Conductive Films with Lower Energy Photonically Sintered Copper/tin Ink**

Xiaolian Chen^1,2^, Xinzhou Wu^2^, Shuangshuang Shao^2^, Jinyong Zhuang^2^, Liming Xie^2^, Shuhong Nie^2^ , Wenming Su^*,2^, Zheng Chen^*,2^, Zheng Cui^2^

^1^ School of Nano-Tech and Nano-Bionics, University of Science and Technology of China, Hefei, 230026, People’s Republic of China.

^2^ Printable Electronics Research Centre, Suzhou Institute of Nano-Tech and Nano-Bionics, Chinese Academy of Sciences, Suzhou, 215123, People’s Republic of China.

*E-mail:wmsu2008@sinano.ac.cn, zchen2007@sinano.ac.cn

**Schematic illustration of the hybrid printing for metal-mesh TCFs on PET substrate:**

**
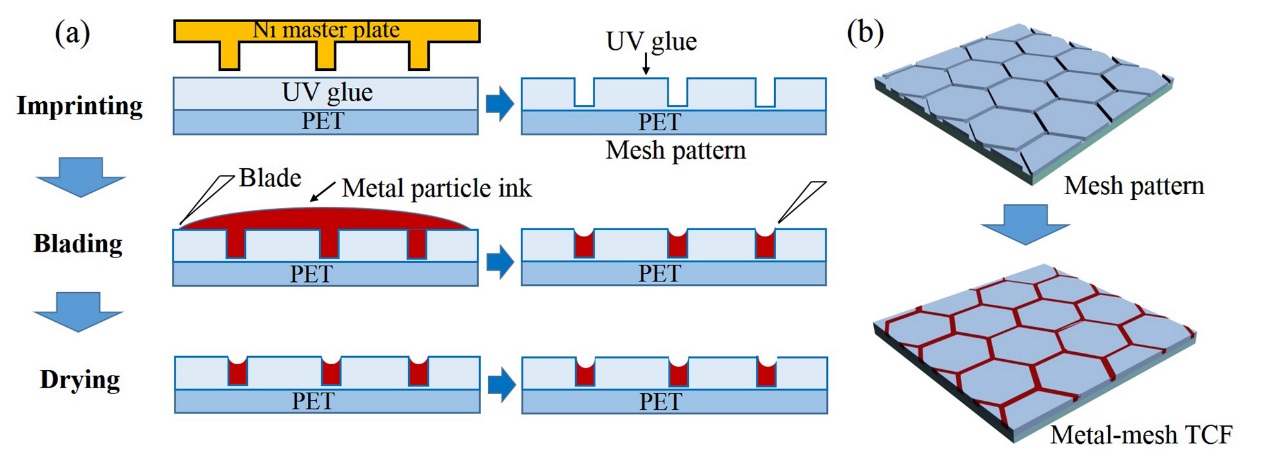
**

**Figure S1.** Schematic illustration of the hybrid printing for metal-mesh TCF on PET substrate: (a) Cross profile schematics for fabrication process of metal-mesh TCE; (b) 3D schematics of fabrication process.

**The sintering energy density of the lamp system:**

The energy density output for the lamp system is determined by the formula:

J= (V/3060)^2.3*0.938*t/S (S1)

J is energy density which is irradiated from flash lamp to the film, V and t are pulse voltage and pulse time, S is the irradiated area of xenon flash lamp (145.2 cm^2^). The formula was provided by the instruction manual of the lamp supplier.

**The optical microscope images and height profiles of metal-mesh TCFs:**

Figure S2a and S2b showed metal-mesh TCFs are in good condition before sintering. The trenches were filled with Cu and Cu/Sn ink. According to height profiles, the metal grid thickness for Cu and Cu/Sn were little different, about 2.5 - 2.7 μm. After sintering by 7.99 J/cm^2^, the Cu actually protruded out of the surface due to heat induced stress while there was no noticeable protruding for Cu/Sn metal mesh TCF (Figure S2c and S2d).


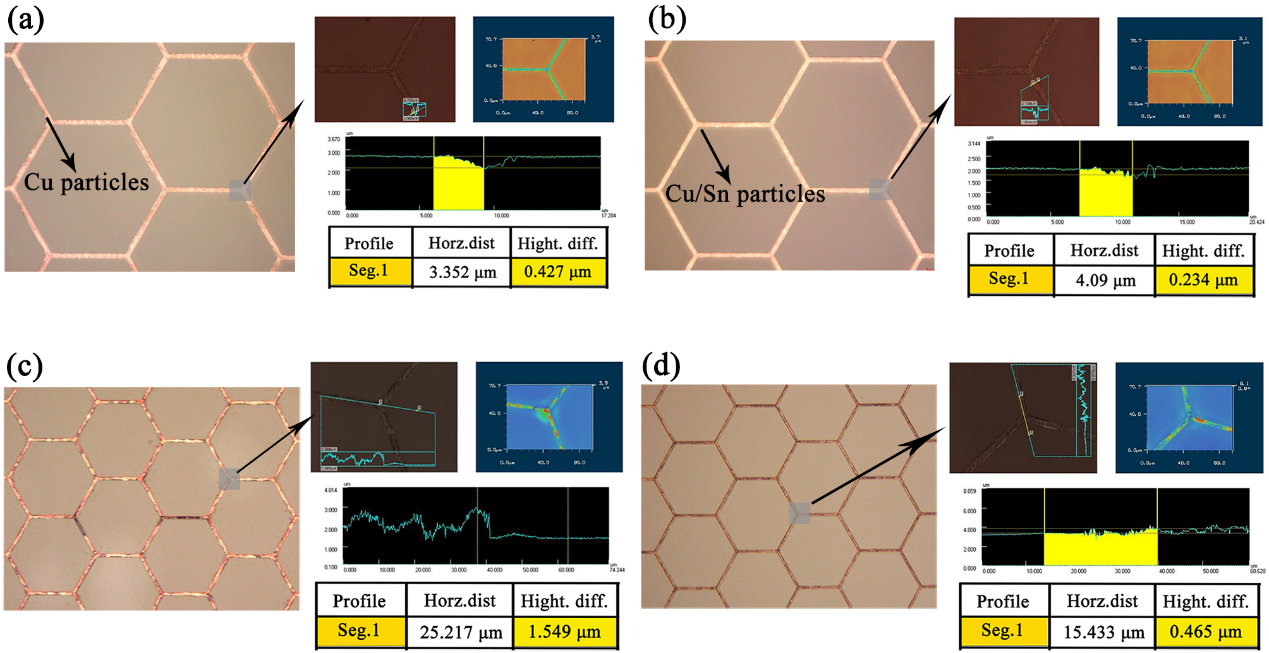


**Figure S2**. The optical microscope images and height profiles of metal-mesh TCFs: Before sintering (a) Cu and (b) Cu/Sn of 1:1; after sintering by 7.99 J/cm^2^ (c) Cu and (d) Cu/Sn of 1:1.

**The transmittance of mesh pattern and metal-mesh TCFs:**

Figure S3 showed the transmittance of 84% at 550 nm for Cu and Cu/Sn metal-mesh TCF sintered by 8.66 J/cm^2^, and 91% for mesh pattern without filling particle ink.


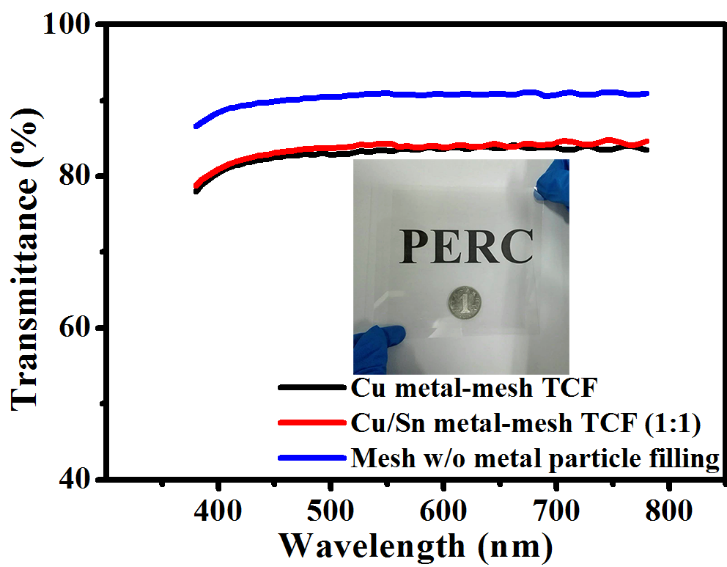


**Figure S3**. The transmittance of mesh without filling metal ink and metal-mesh TCFs with filing particle ink; the inset showed the optical image of Cu metal-mesh TCF.

**SEM images of Cu and Sn particles:**

SEM images showed Cu particles have a spherical shape with an average diameter of 150 nm ± 30 nm while the commercial Sn particles are in the range of 100 - 300 nm in diameter.


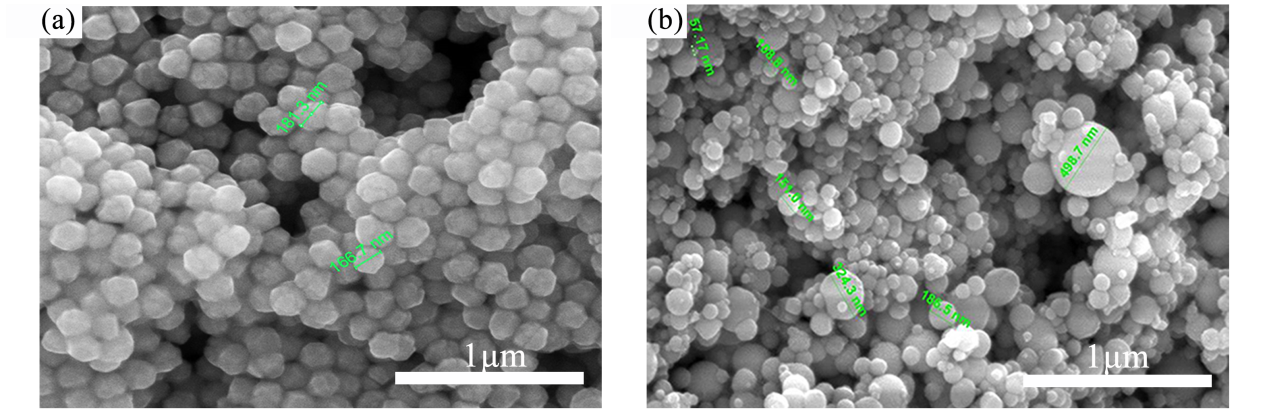


**Figure S4**. SEM images of particles: (a) Cu particles; (b) Sn particles.

**Optical microscopy images of mesh pattern with hexagonal structure on PET substrate:**

The line width and the depth of trenches are 3.5 μm and 3 μm, respectively. At the same time, the side length of hexagon is 74 μm.


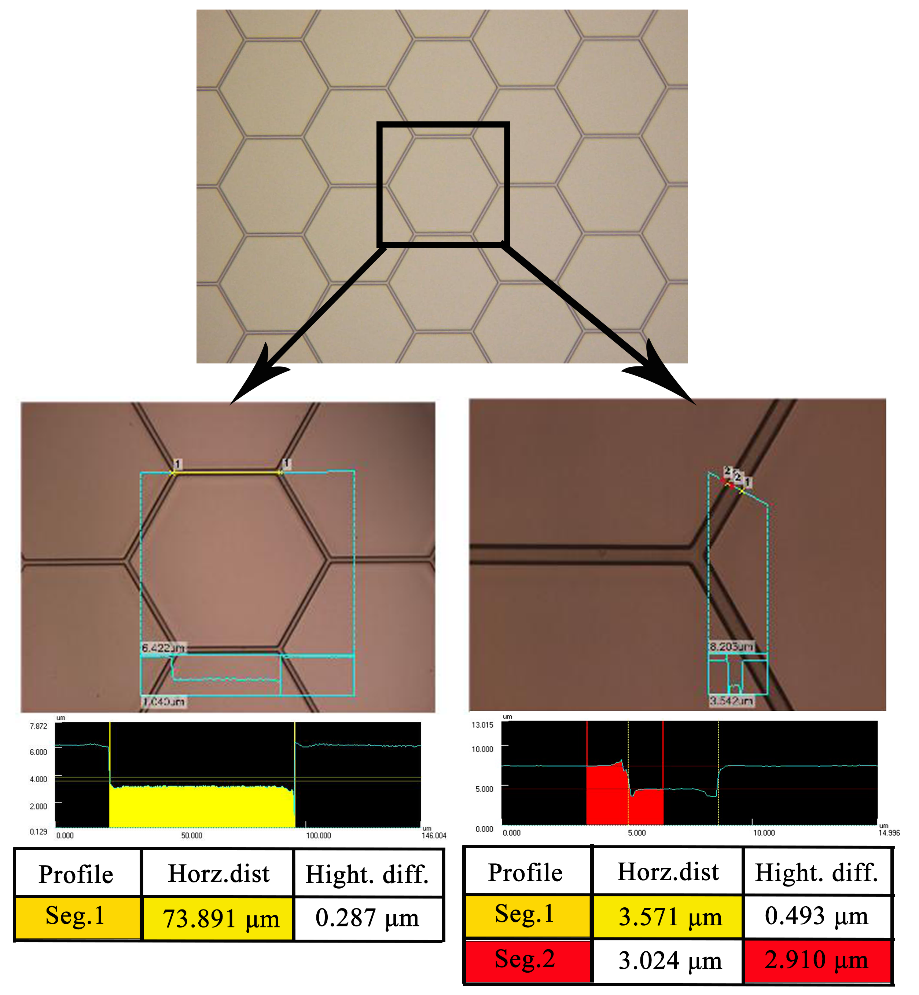


**Figure S5**. The optical microscopy images of the mesh pattern with hexagonal trench structure on PET.

**The cross-sectional profiles of metal films:**

The cross-sectional profile analysis using step profiler was conducted (Figure S6). It was measured from a to b (the inset of Figure S6a) and from c to d (the inset of Figure S6d). The Cu and Cu/Sn films before sintering had the same average thickness, ~ 6 μm (Figure S6a – S6c). Meanwhile, the thickness of the Cu and Cu/Sn films decreased to ~ 5 μm after the 4.69 J/cm^2^ flash light-sintering process (Figure S6d – S6f).


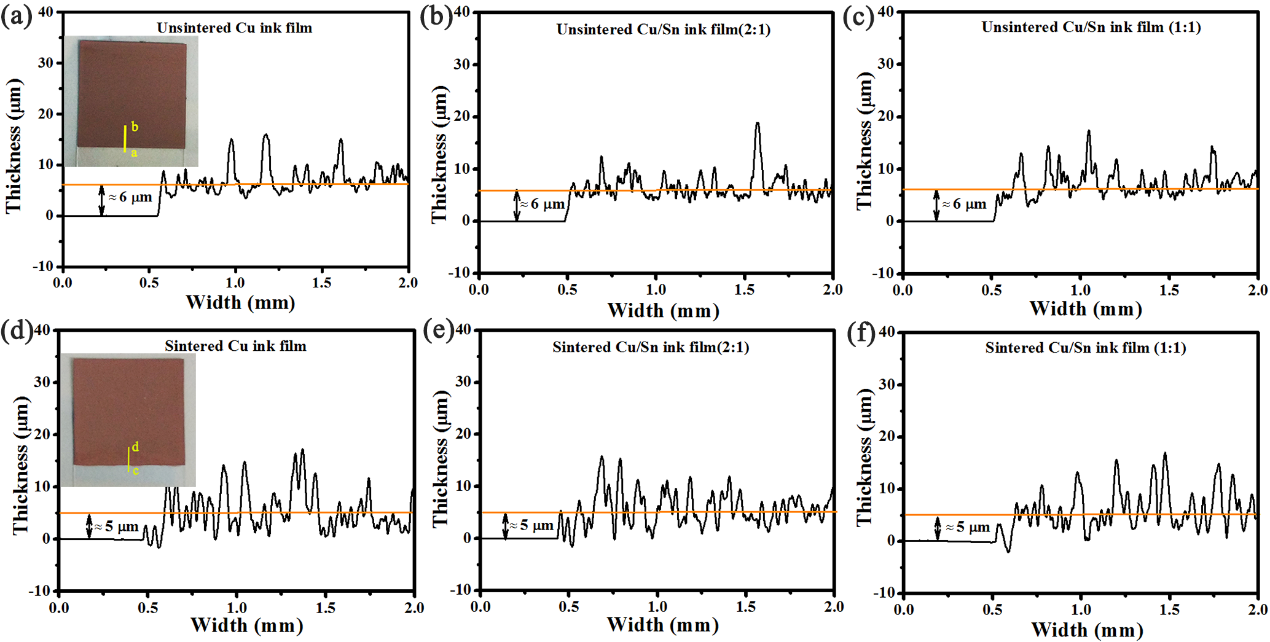


**Figure S6**. Surface profiles of metal films: before sintering (a) Cu and the photograph image of screen-printed film on PET substrate(the inset), (b) Cu/Sn of 2:1 and (c) Cu/Sn of 1:1; after sintering by 4.69 J/cm^2^; (d) Cu and the photograph image of sample (the inset), (e) Cu/Sn of 2:1 and (f) Cu/Sn of 1:1.

**Elemental mapping of Cu/Sn film (1:1):**

**
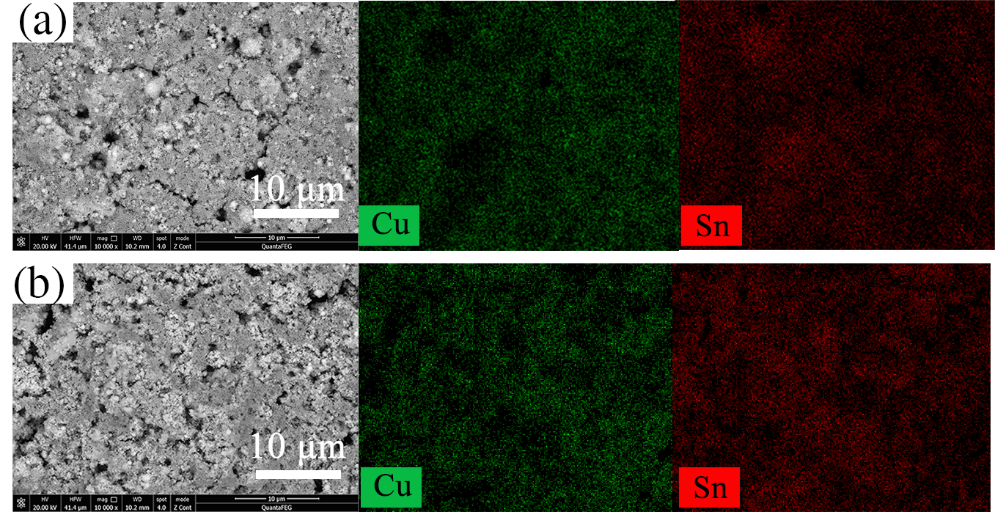
**

**Figure S7.** SEM image and elemental mapping for Cu/Sn films by screen printing (1:1): (a) Before Sintering; (b) After sintering by 4.69 J/cm^2^.

**The absorption spectrum of Cu and Sn:**

Cu and Sn particle inks was diluted 500 times with alcohol. The absorption spectra was analyzed with a UV-visible spectrometer (Lambda 750, PerkinElmer).

**
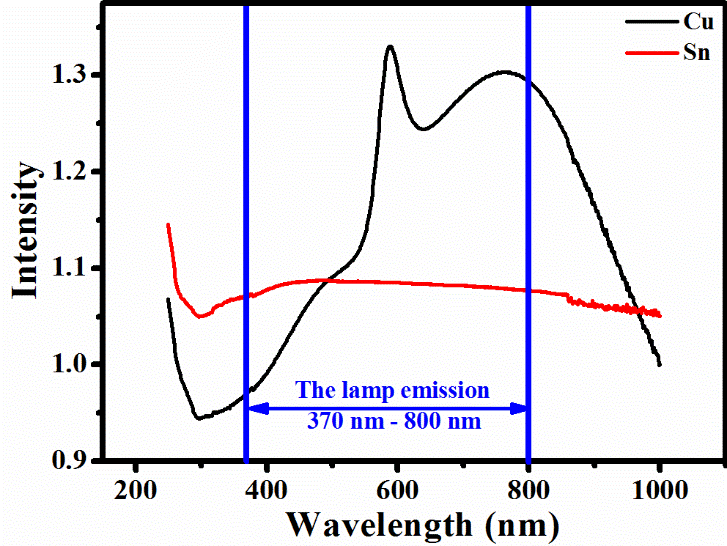
**

**Figure S8**. The UV–vis absorption spectrum of Cu and Sn.

**DSC curves:**

The solid contents for various inks were scraped off the samples by screen printing. As shown in Figure S9, the DSC curves of Sn and mixed Cu/Sn ink presented the endothermic peak around 230 ^o^C while it didn’t exist in the Cu ink.


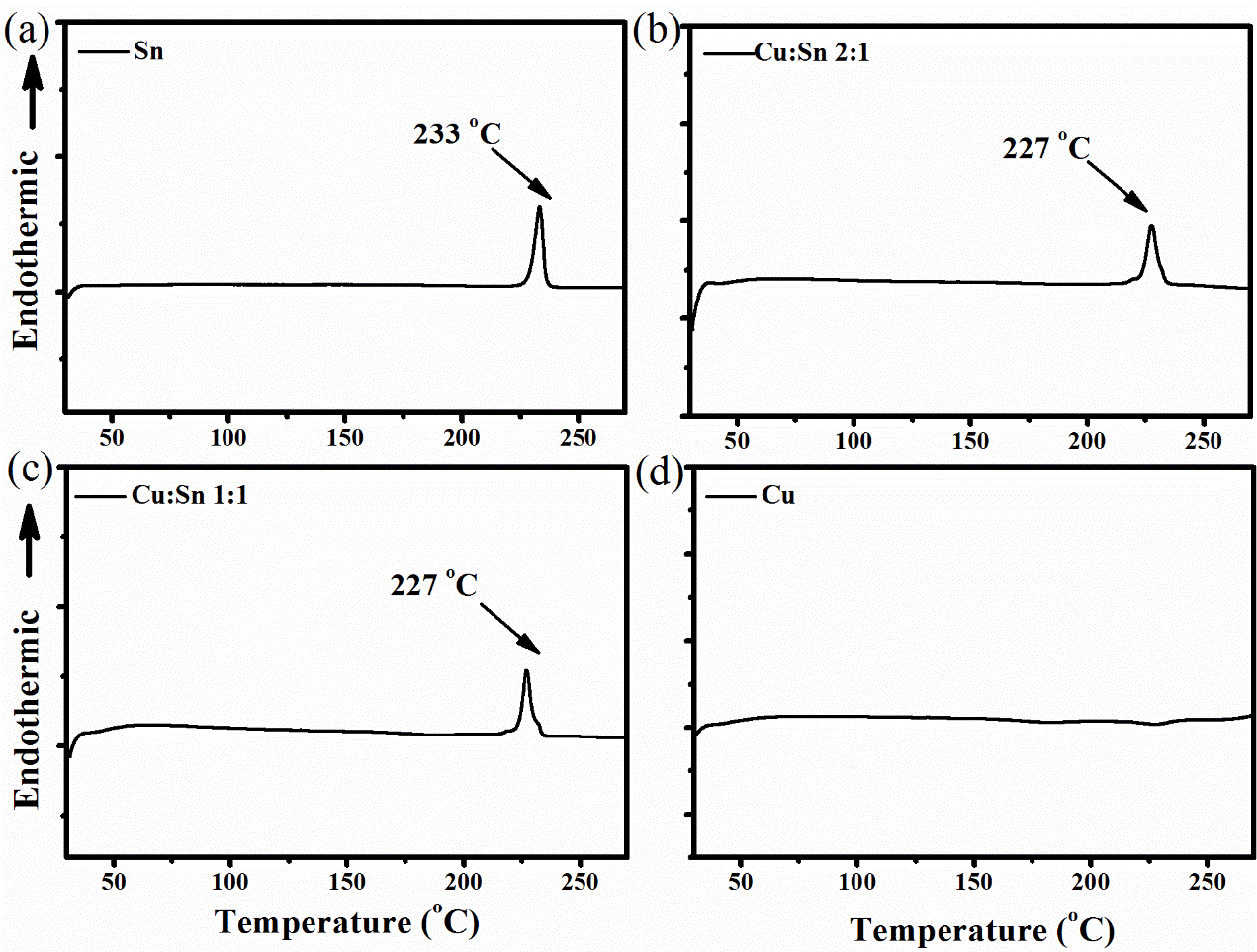


**Figure S9.** DSC curves of (a) Sn particles, Mixed Cu/Sn particles of (b) 2:1 and (c) 1:1, and (d) Pure Cu particles.

**The sintering defects of Cu metal-mesh transparent conductive film:**

There were many sintering defects on Cu metal-mesh TCF under high energy, for example, the mesh structure was destroyed and many Cu lines were fractured.

**
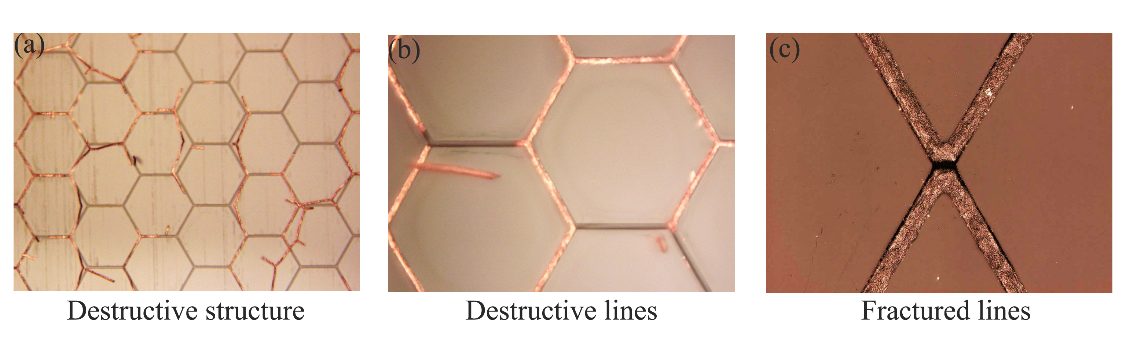
**

**Figure S10.** The sintering defects on Cu metal-mesh TCFs sintered by high energy density: (a) Destructive grid structure; (b) Destructive Cu lines; (c) Fractured Cu lines.

**The optical microscope images for different quality of metal-mesh TCF:**

The quality of film of metal-mesh TCF was evaluated using the grade scheme. Grade 1 indicates perfectly intact structures in all parts of the test pattern. Grade 2 indicates minor fractured lines. Grade 3 indicates destructive lines and structures below 5% of the whole area. Grade 4 indicates destructive lines and structures at 5% to 20% of the whole area. Grade 5 indicates major damage that make it difficult to measure the sheet resistivity.


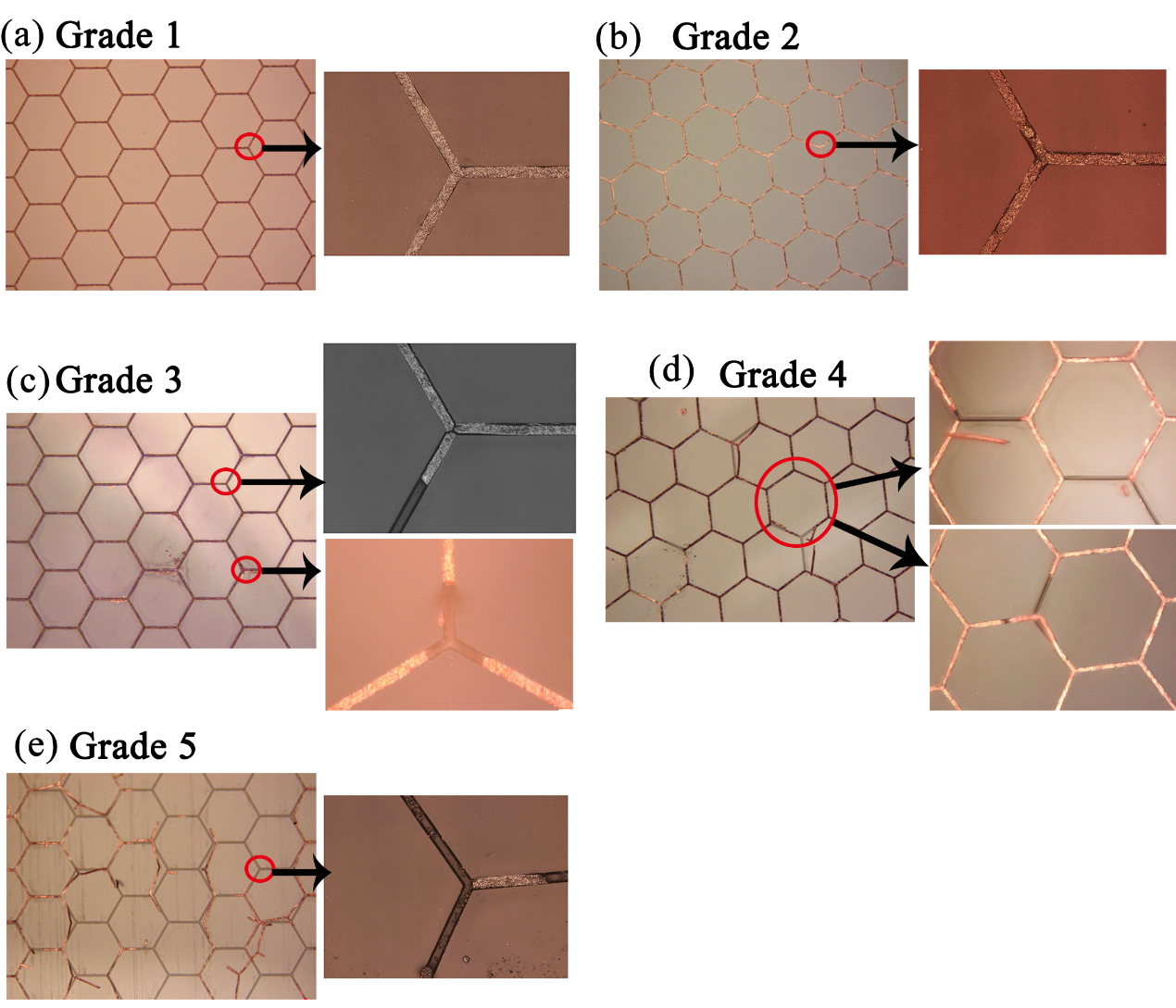


**Figure S11.** The optical microscope images for different quality of metal-mesh TCF: (a) Grade 1; (b) Grade 2; (c) Grade 3; (d) Grade 4; (e) Grade 5.
